# Supplementary material for: Plasma Small Extracellular Vesicles with Complement Alterations in GRN/C9orf72 and Sporadic Frontotemporal Lobar Degeneration
Source: Cells. 2022 Jan 30;11(3):488. doi: 10.3390/cells11030488 (PMC8834212; doi:10.3390/cells11030488)
Supplement: Supplementary file 1 [file cells-11-00488-s001.zip › cells-1514005-supplementary/Supplementary_TableS1.pdf]

Supplementary Material

1 Supplementary Tables

Supplementary Table S1. FTLD causing mutations in *GRN* mutation carriers included in the study.

N. of patients	Gene	Mutation	Aminoacidic Variation	Type of variant
32	GRN	c.813-816delCACT	p.Leu271LeufsX10	Null
5	GRN	c.C1021T	p.Gln341X	Null
2	GRN	c.A211G	EX0-5′ splicing	Null
1	GRN	c.C328T	p.Arg110X	Null
1	GRN	c.468_474delCTGCTGT	p.Cys157LysfsX97	Null
1	GRN	c.829_830delCA	p.Thr276SerfsX7	Null
1	GRN	c.829_834del	p.Thr278SerfsX7	Null
1	GRN	c.445_446delTG	p.Cys149LeufsX10	Null
1	GRN	c.708+6_708+9delTGAG	Intronic variant at splice site	Null
2	GRN	c.G314A	p.Cys105Tyr	Missense
1	GRN	c.T415C	p.Cys139Arg	Missense
1	GRN	c.G893A	p.Arg298His	Missense

GRN reference: NM_002087.4
