# Supplementary figures and images for: Plasma Small Extracellular Vesicles with Complement Alterations in GRN/C9orf72 and Sporadic Frontotemporal Lobar Degeneration
Source: Cells. 2022 Jan 30;11(3):488. doi: 10.3390/cells11030488 (PMC8834212; doi:10.3390/cells11030488)

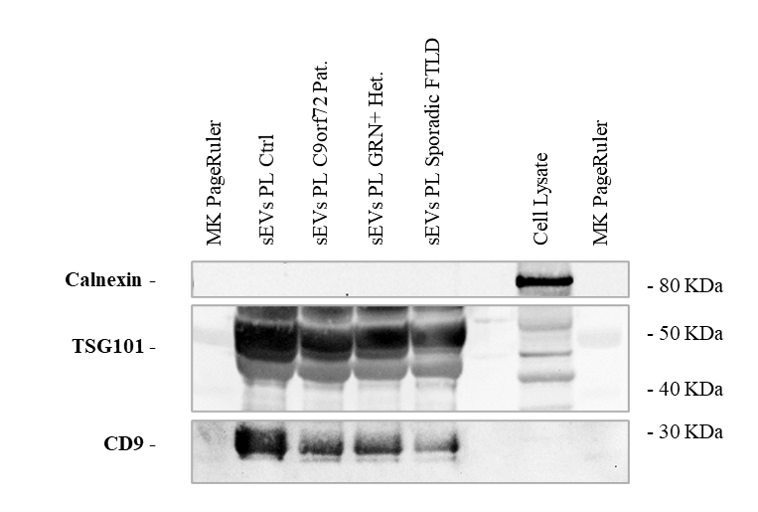

Supplement: Supplementary file 1 [file cells-11-00488-s001.zip › cells-1514005-supplementary/Supplementary_FigureS1_RevisedVersion.tif]
